# The whole-genome sequencing in predicting *Mycobacterium tuberculosis* drug susceptibility and resistance in Papua, Indonesia

**DOI:** 10.1186/s12864-021-08139-3

**Published:** 2021-11-22

**Authors:** Yustinus Maladan, Hana Krismawati, Tri Wahyuni, Ratna Tanjung, Kamla Awaludin, Kholis Abdurachim Audah, Arli Aditya Parikesit

**Affiliations:** 1 Center for Papua Health Research and Development, Papua, Indonesia; 2 Jayapura Regional Health Laboratory, Papua, Indonesia; 3 Department of Biomedical Engineering, Swiss German University, Tangerang, Indonesia; 4 Department of Bioinformatics, School of Life Sciences, International Institute for Life Sciences (I3L), Jakarta, Indonesia

## Abstract

**Background:**

Tuberculosis is one of the deadliest disease caused by *Mycobacterium tuberculosis*. Its treatment still becomes a burden for many countries including Indonesia. Drug resistance is one of the problems in TB treatment. However, a development in the molecular field through Whole-genome sequencing (WGS) can be used as a solution in detecting mutations associated with TB- drugs. This investigation intended to implement this data for supporting the scientific community in deeply understanding any TB epidemiology and evolution in Papua along with detecting any mutations in genes associated with TB-Drugs.

**Result:**

A whole-genome sequencing was performed on the random samples from TB Referral Laboratory in Papua utilizing MiSeq 600 cycle Reagent Kit (V3). Furthermore, TBProfiler was used for genome analysis, RAST Server was employed for annotation, while Gview server was applied for BLAST genome mapping and a Microscope server was implemented for Regions of Genomic Plasticity (RGP). The largest genome of *M. tuberculosis* obtained was at the size of 4,396,040 bp with subsystems number at 309 and the number of coding sequences at 4326. One sample (TB751) contained one RGP. The drug resistance analysis revealed that several mutations associated with TB-drug resistance existed. In details, mutations of *rpoB* gene which were identified as S450L, D435Y, H445Y, L430P, and Q432K had caused the reduced effectiveness of rifampicin; while the mutases in *katG* (S315T), *kasA* (312S), *inhA* (I21V), and Rv1482c-*fabG1* (C-15 T) genes had contributed to the resistance in isoniazid. In streptomycin, the resistance was triggered by the mutations in *rpsL* (K43R) and *rrs* (A514C, A514T) genes, and, in Amikacin, its resistance was led by mutations in *rrs* (A514C) gene. Additionally, in Ethambutol and Pyrazinamide, their reduced effectiveness was provoked by *embB* gene mutases (M306L, M306V, D1024N) and *pncA* (W119R).

**Conclusions:**

The results from whole-genome sequencing of TB clinical sample in Papua, Indonesia could contribute to the surveillance of TB-drug resistance. In the drug resistance profile, there were 15 Multi Drugs Resistance (MDR) samples. However, Extensively Drug-resistant (XDR) samples have not been found, but samples were resistant to only Amikacin, a second-line drug.

**Supplementary Information:**

The online version contains supplementary material available at 10.1186/s12864-021-08139-3.

## Background

Many innovations in tuberculosis (TB), as one of the top killer infectious diseases in many countries, were performed in diagnostic, transmission, drug resistance, drug therapy, prevention, and control programs. Indonesia is one of eight countries that accounts for two-thirds of the world’s total TB cases, with a percentage of 8.5% [1]. Drug-resistant TB is a problem that needs extra attention when controlling tuberculosis [2]. Testing for susceptibility and resistance to anti-TB drugs can be carried out conventionally with the culture method. However, this method is less effective because of its time-consuming. Thus, a faster method is needed, such as a molecular approach [1].

Next-generation sequencing (NGS) is a powerful tool for improving the management and control of tuberculosis [3, 4]. Whole-genome sequencing (WGS) data from NGS can be used to quickly and accurately detect mutations associated with anti-TB drug resistance in clinical specimens [3].

The first complete TB genome was published in 1998 from the H37Rv strain [5]. After that, an advanced biomedical research was performed to complete many gaps of this slow-growing bacteria. Using NGS technologies, performing WGS in TB seems applicable to a wide range of clinical scenarios, even nationally. The whole bacterial genome sequencing can provide comprehensive data such as drug susceptibility and resistance prediction, epidemiological analysis, and research [6–8]. In addition, the existence of the WGS dataset allows researchers to assess genetic diversity across the genome looking for markers of selective stress. Selection can be measured by associating the ratio of nonsynonymous genetic change to synonymous change (dN/dS), in which a dN/dS is more remarkable than others will be considered to reflect positive selection for the increased diversity [4].

WGS technologies allow the generation of the drug susceptibility in about 8–9 days, while conventional drug susceptibility tests need more than 14 days to perform the result. This technology becomes a valuable tool for monitoring antibiotic resistance which lead to the optimization of field intervention to prevent the broader spread of TB drug resistance. WGS can be carried out directly from the clinical samples because the online tools are available for data interpretation. Thus, the WGS data can be used for epidemiological analysis and research purposes [6, 8]. University Medical Center Groningen (UMCG) had implemented these technologies for the outbreak management, pathogen characterization, and surveillance, as well as the rapid identification of bacteria using the 16S–23S rRNA region, taxonomy, metagenomic approaches to clinical samples, and the determination of the zoonotic micro-organisms transmission from animals to humans [9].

Besides the technology to execute the WGS on TB, the development of many bioinformatics tools also enhances the increasing use of WGS, especially in data analysis. Recently, some approaches to interpret the results from the NGS machine have availed.

Comprehensive and robust open-source packages for the standardized NGS analysis also has appeared, such as UGENE [10]. There is many tools existed for genome analysis of WGS result. Some are available online such as Microscope for microbial comparative genome analysis and manual functional annotation [11]. The Microscope has the data-exploring features with many beneficial functionalities, such as allowing the users to perform (complex) queries, comparative genomics studies, and metabolic analyzes. It also provides the summary views of statistics and information for each genome as a whole. Usually, a complete Rapid Annotation using Subsystems Technology (RAST) was added for the same function. RAST is a fully automated service for annotating the complete or nearly complete bacterial and archaeal genomes. It gives high-quality genome annotations for these genomes across the whole phylogenetic tree [12, 13]. For drug resistance analysis, the webserver of TB-Profiler allows users to analyze *M. tuberculosis* WGS data to predict its lineage and drug resistance [14, 15].

Currently, TB Control has become one of the national priorities of health development in Indonesia. Although Indonesia has made remarkable progress over the last decades, this disease remains becoming one of the country’s top four causes of death. The easternmost part of Indonesia, especially Papua, was the most significant TB contributor in Indonesia lacking of studied data due to its geographical challenges. This paper presented WGS results from the clinical samples of pulmonary TB in Papua with the intention to contribute this data in supporting the scientific community to profoundly understand the epidemiology and evolution of TB in Papua.

## Results

A total of 20 samples were sequenced, only 19 samples had good data quality. WGS was utilized in genotyping 19 samples of *M. tuberculosis*. The results of NGS reading compared to *M. tuberculosis* H37Rv as a reference strain are displayed in Table 1.

**Table 1:** Comparison between H37Rv and *M. tuberculosis* TB751 genomes from Papua

Genome	***M. tuberculosis*** TB751 from Papua	H37Rv (Reference)
Genome size	4,396,040	4,411,532
Number of subsystems	309	389
Number of coding sequences	4326	3928
Number of RNAs	47	60

**Fig. 1:**
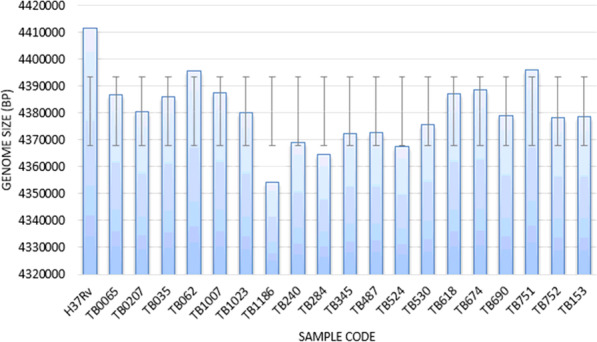
Genome Size of 19 *M. tuberculosis* samples from Papua (SD: 12,685.32). *M. tuberculosis* TB751 had the largest genome size with 4,396,040 bp. *M. tuberculosis* TB751 has a significant difference of 15,492 bp with H37Rv

Data analysis results exhibited that the biggest genome of *M. tuberculosis* in Papua strain was 4,396,040 bp and the smallest was 4,354,210 bp. The genome size for H37Rv was 4,411,532 base pairs (bp) with G + C 65.6% [5]. The standard deviation (SD) obtained was 12,685.32 which indicated a significant difference in the sample genome size is less than 10% (Fig. 1).

**Fig. 2:**
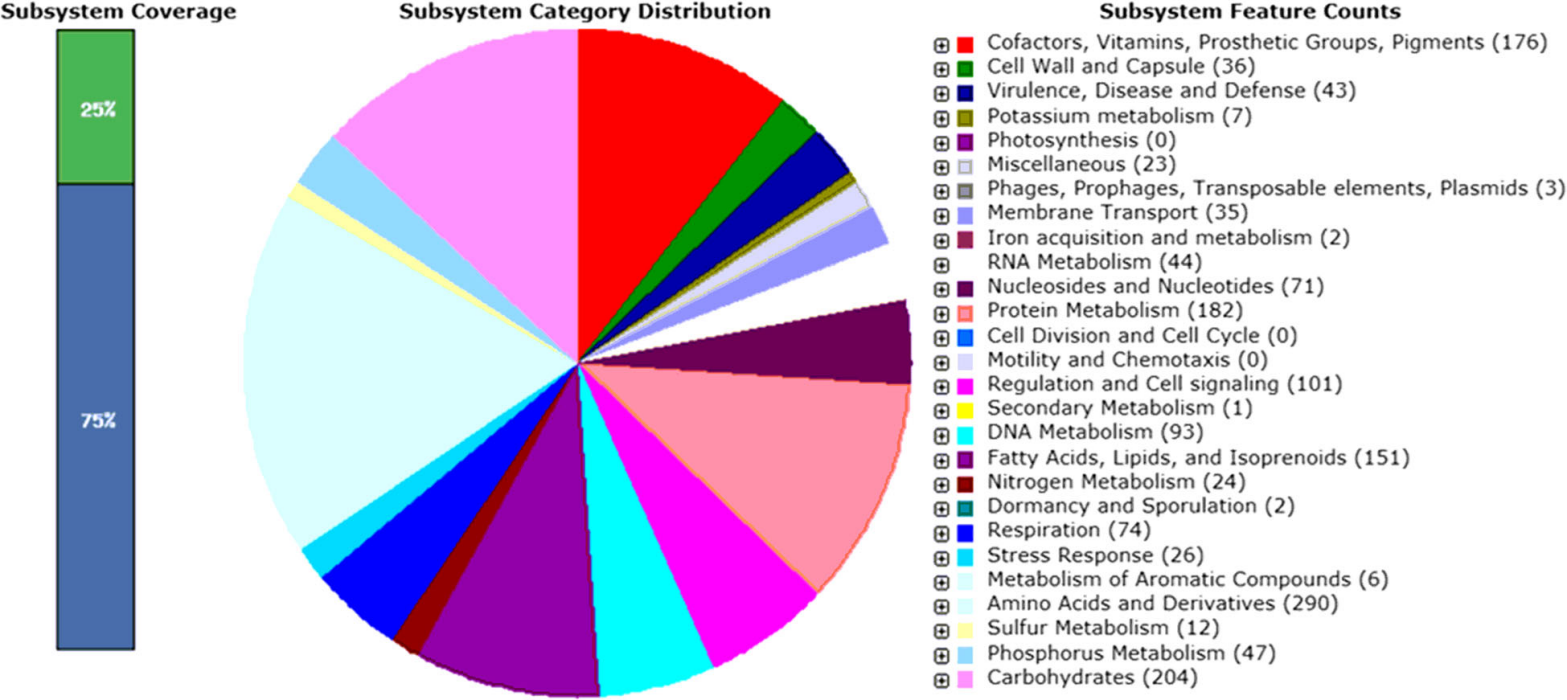
Subsystem distribution of *M. tuberculosis* Papua strain (based on RAST annotation server). In subsystem coverage, 25% is indicated in subsystem and 75% is not in the subsystem. The most genome ontology subsystems are amino acids and derivatives which are accounted for 290

The subsystem distribution of *M. tuberculosis* Papua strains was analyzed using the RAST server and visualized with the SEED server. RAST server identified protein-encoding, rRNA, and tRNA genes; assigned functions to the genes, and predicted which subsystems were represented in the genome. The genome that had been annotated by RAST server can be browsed in the comparative environment of SEED-Viewer. SEED served to provide consistent and accurate genome annotations based on the integration of genome data from genome databases, web front end, API, and server scripts [12, 13]. The sample used was TB751 because it had a larger genome size than the other samples. Largest genome selection was intended to minimize the existed gaps in the genome. The functional classification exhibited that amino acids and derivatives had a larger percentage in *M. tuberculosis* subsystem (290). In addition to amino acids and their derivatives, carbohydrates, cofactors, vitamins, fatty acids, and lipids also possessed a large percentage of the subsystem (Fig. 2). Most of the genes involved in amino acid biosynthesis were highly conserved in all Mycobacterium species [16]. This proved that amino acids had an essential function for pathogens including *M. tuberculosis* [17].

The number of subsystems in *M. tuberculosis* TB751 from Papua was 309 which was 80 points different compared to H37Rv. Meanwhile, compared to the number of coding sequences with H37Rv, it was 398 point different. Meanwhile, the gap number of RNAs was 13 (Table 1). *M. tuberculosis* with Papua strain has a G + C content at 65.6%. These discrepancies could be feasible due to the difference in the annotation coverage strategies and in the instrument utilization.

**Fig. 3:**
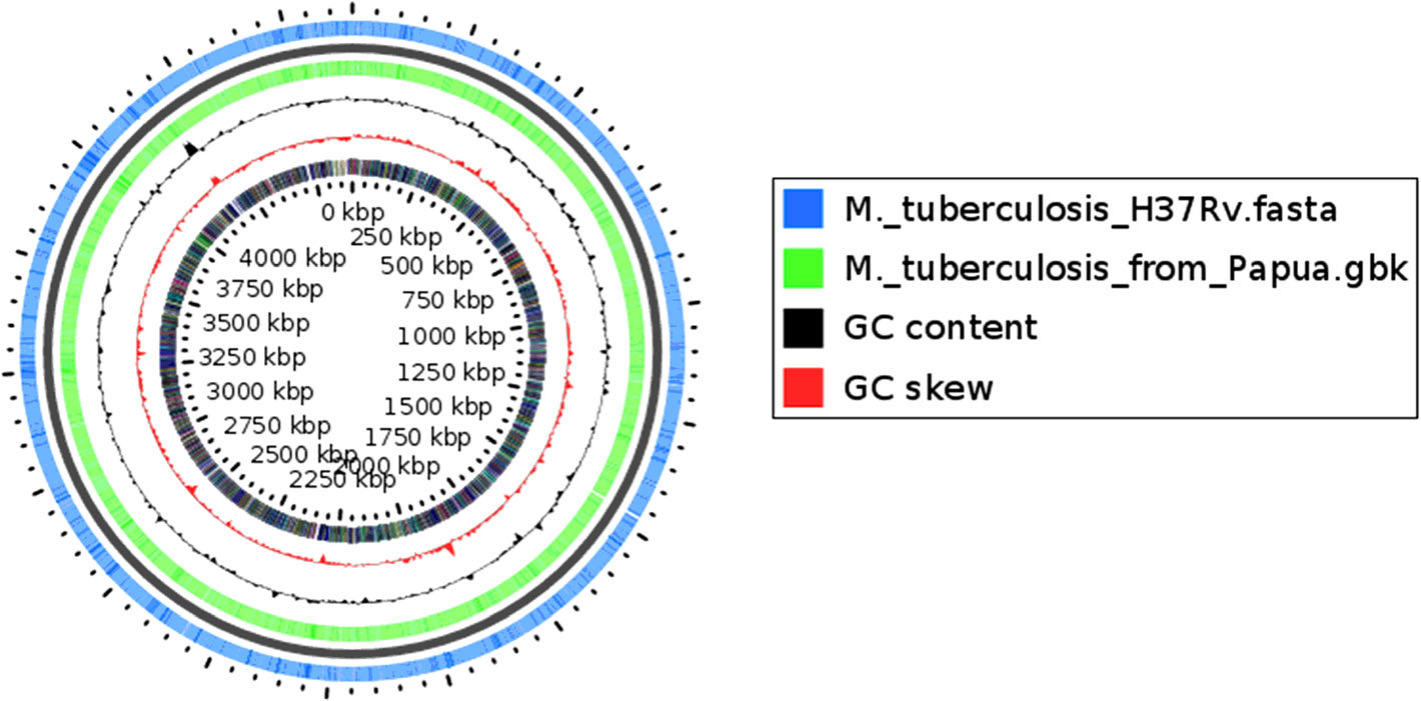
The BLAST Atlas of *M. tuberculosis* TB751 Papua strain. BLAST was performed to the coding sequences (CDS) regions in the reference genome against CDS regions in the query genomes and its top hits were rendered in a genome map using GView. The inner-most slot on the map (black) shows CDS regions on the reference genome. The two outer slots (green and blue) display regions of *M. tuberculosis* H37Rv (blue) and *M. tuberculosis* from Papua (green)

The BLAST Atlas of *M. tuberculosis* Papua strain was created using the Gview server [18]. BLAST atlas was utilized to compare the genome of *M. tuberculosis* TB751 from Papua with H37Rv. *M. tuberculosis* TB751 from Papua was different with H37Rv at 15,492 bp. However, CDS of *M. tuberculosis* TB751 had an almost similar distribution as the H37Rv genome (Fig. 3).

**Fig. 4:**
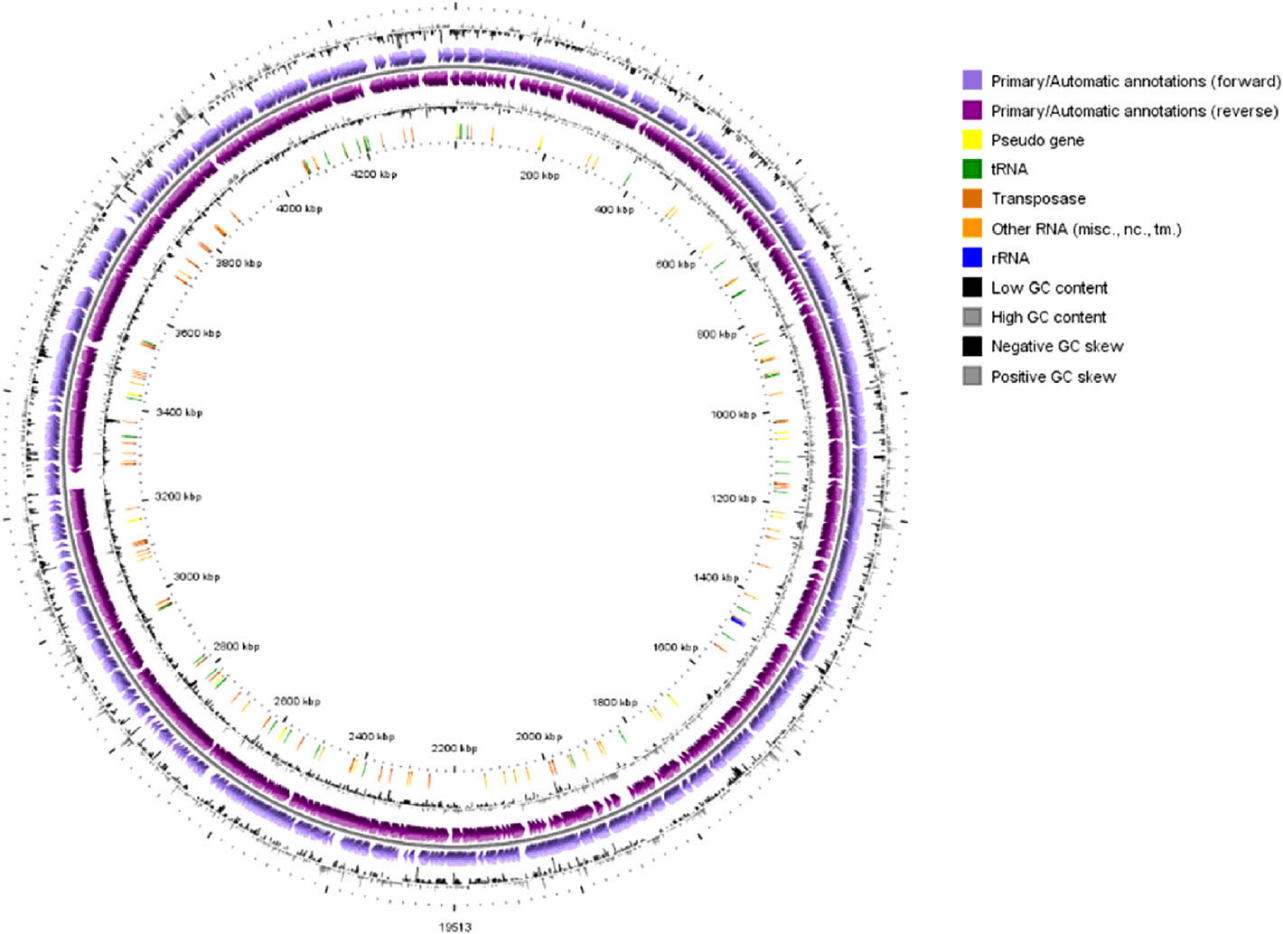
Genome information of *M. tuberculosis* TB751. Circles display (from the outside): 1. Gene GC percentage deviation (gene GC% - genome mean GC%), 2. Predicted CDSs transcribed in the clockwise direction (violet), 3. Predicted CDSs transcribed in the counterclockwise direction (purple), 4. Gene GC skew (G-C/G + C), 5. rRNA (blue), tRNA (green), misc. RNA (orange), transposable elements (brown) and pseudogenes (yellow)

According to the Microscope server analysis, TB751 has an average CDS length of 929.12 bp, average intergenic length at 121 24, protein coding density at 91.32%, and nosferatu repeated regions at 8.63%. TB751 contained 8 pseudogenes. The genomic information of TB751 consists of 4483 genomics objects, 45 tRNAs, 3 RNAs, 36 misc_RNAs, 1 tmRNA, 0 ncRNA. Meanwhile, ribosomal RNA consists of, 1 ribosomal RNA 16S_rRNA, 1 RNA 23S_rRNA and 1 RNA 5S_rRNA. The genomics information of TB751 is presented in a circular model using CGview [19] (Fig. 4).

The comparison of the genome of *M. tuberculosis* TB 751 and *M. tuberculosis* H37Rv was carried out by employing a Microscope Server [11]. The RGF Finder on Microscope servers was able to search for any potential horizontally transferred genes (HGT) which were gathered in the genomic regions (Region of Genomic Plasticity). A region is considered RGP if a break must span at least 5 kb and must not contain more than 2 genes in the synteny. After that, the algorithm scans RGP for any HGT features (tRNA hotspot and/or mobility genes). Alien Hunter [20] which is an Interpolated Variable Order Motif (IVOM) has similar function as Score-Based Identification of Genomic Islands-Hidden Markov Models (SIGI-HMM) [21] to capture other kinds of signals of the query sequence. A microscope incorporated with an IVOM or a SIGI-HMM will predict the overlapping regions but not the ones overlapped with RGP. In fact, *M. tuberculosis* TB751 contained an RGP. In the RGP of *M. tuberculosis* TB751, there were no overlapping regions based on the examination with SIGI and IVOM. RGP of *M. tuberculosis* 751 revealed that two regions did not exist in *M. tuberculosis* H37Rv. Both regions, in fact, were of a miscellaneous RNA (miscRNA) type (Table 2).

**Table 2:**
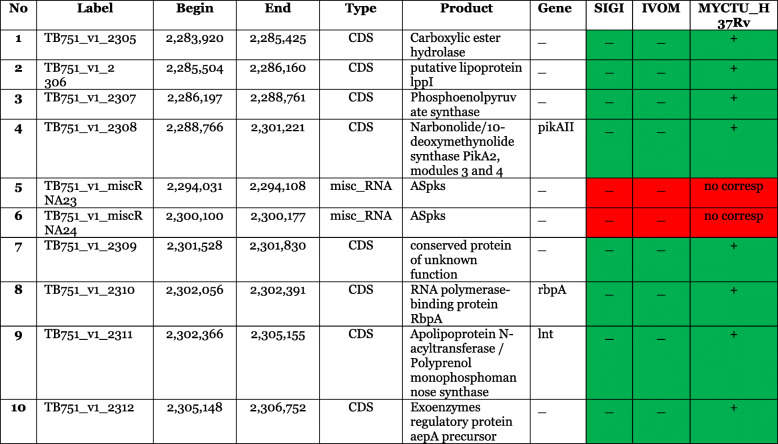
Comparison of Regions of Genomic Plasticity in *M. tuberculosis* Papua strain and *M. tuberculosis* H37Rv using RGP Finder. Green: similar gene in the compared genome above cut-off value. Red: no similarity above the identity cut-off value. Red with ‘no corresponding’ text: no similarity at all.

**Fig. 5:**
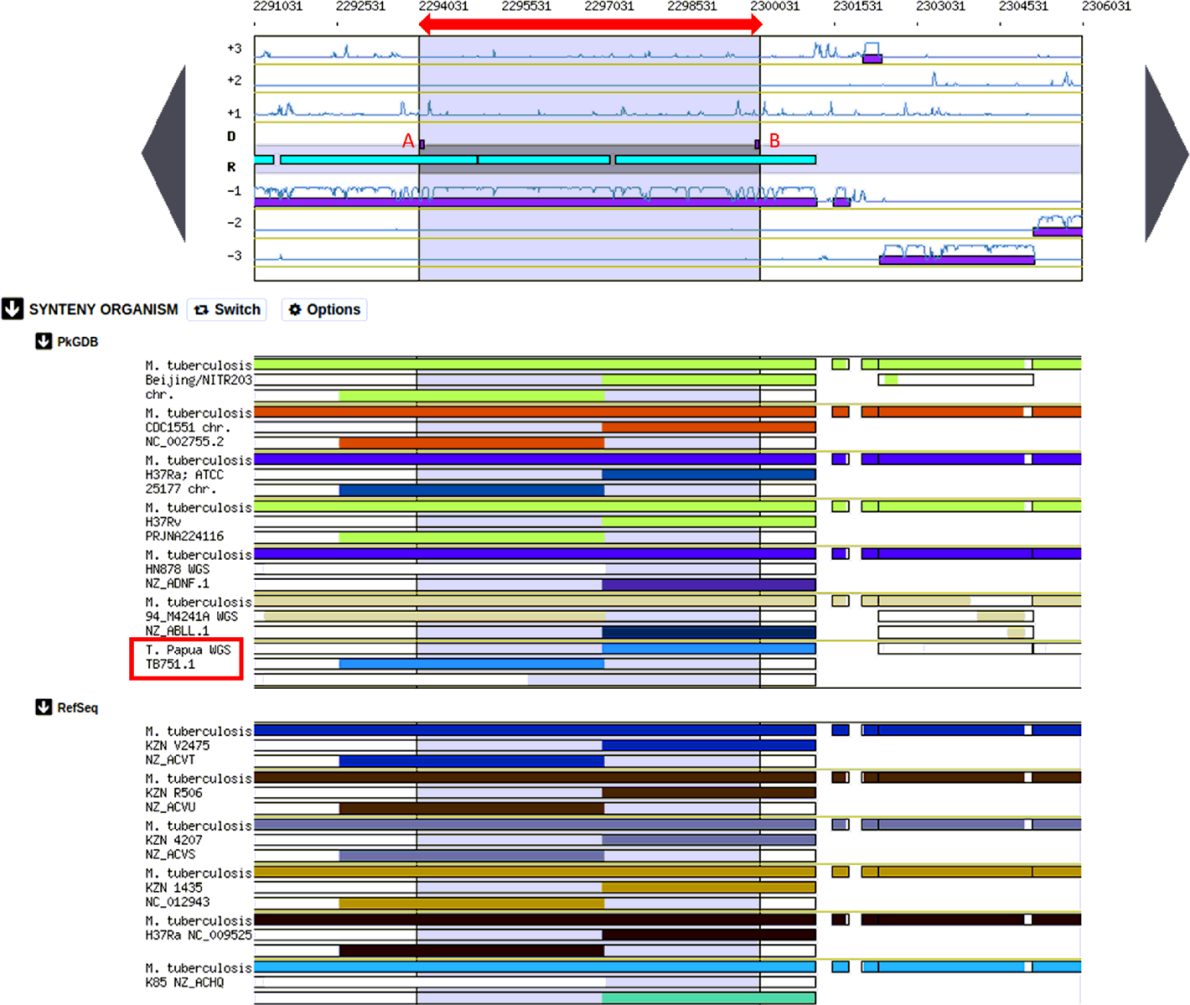
Genome cartographic map in the region of misc_RNA. A: TB751_v1_miscRNA23; B: TB751_v1_miscRNA24. Misc_RNA is markerd in purple and located in the middle, green Tosca is the repeat regions. The upper part of the window details the Coding Sequences (CDSs) that have been predicted for reading frames + 1, + 2 and + 3 in the current region; the middle part indicates the position of RNA objects (rRNA, tRNA, misc_RNA) as well as repeated regions (as turquoise rectangles) if any have been detected; and the bottom part of the window shows CDSs that have been predicted for reading frames − 1, − 2 and − 3. The color of the rectangles reflects the illustrating synteny conservation, to the exception of the white color (*M. tuberculosis* Beijing, *M. tuberculosis* CDC1551, *M. tuberculosis* H37Ra, *M. tuberculosis* H37Rv PRJNA224116, *M. tuberculosis* HN878, *M. tuberculosis* 94_M4241A, and *M. tuberculosis* TB751). Rectangles filled with white color indicate homologs which don’t belong to any synteny group (*M. tuberculosis* HN878 and *M. tuberculosis* TB751). The same color on 2 synteny map rows does not indicate any synteny relationship

Miscellaneous RNA is shown in the middle part of the genome map (Fig. 5). The locus coordinate of the genome annotations in TB751_v1_miscRNA23 began at 2,294,031 and ended at 2,294,108, while for TB751_v1_miscRNA24, it began at 2,300,100 and ended at 2,294,108. Genome Browser interface visualized and explored a replicon content (cartographic map of the genome) along with the similarity results (synteny maps) obtained and other bacterial genomes available in this study’s PkGDB database Microscope or the complete proteome downloaded from the RefSeq /WGS sections. The synteny maps were calculated for all pairs of genomes from the PkGDB database (first synteny map) or the NCBI databank (second map). They represented the distribution of homologs of the current genome in other genomes from these databases. Each row on the map corresponded to one genome replicon (chromosome or plasmid) whose name is indicated on the left. The homologous gene in the regions between 2,294,031-2,300,177 was the region that encoded the polyketide synthase. Based on the PkGDB data, the area was homologous to *M. tuberculosis* Beijing strain, *M. tuberculosis* CDC1551, *M. tuberculosis* H37Ra, *M. tuberculosis* H37Rv PRJNA224116, *M. tuberculosis* HN878, *M. tuberculosis* 94_M4241A, and *M. tuberculosis* TB751 from Papua. In PkGDB, the region bounded by the gray area was the CDS region that produced polyketide synthase (Fig. 5).

Furthermore, the identification of TB drug resistance both in the first line and in the second line could be done using TBProfiler (Table 3). TBProfiler had the sensitivity in detecting MDR-TB which was 94% with a specificity of 98%. For XDR-TB, the sensitivity was 83% and its specificity was 96% [15]. Multidrug-resistant tuberculosis (MDR-TB) appears if *M. tuberculosis* does not respond to, at least, isoniazid and rifampin. Whereas Extensively drug-resistant TB (XDR-TB) is a form of MDR-TB which is also resistant to two groups of second-line anti-TB drugs [22]. A total of 15 samples obtained had MDR and no XDR was found. In regard to spoligotype family, 19 samples of *M. tuberculosis* from Papua consisted of three spoligotype families, namely East African-Indian (EAI), Latin American-Mediterranean (LAM), and Beijing RD105.

**Table 3:** Resistance phenotype and genotype of matched pairs. Inh (isoniazid); Rif (rifampin); Emb (ethambutol); Str (streptomycin); Pza (pyrazinamide); Amk (amikacin)

Sample Code	Xpert	Spoligo. Family	Inh	Rif	Emb	Str	Pza	Amk	Note
TB0065	R	EAI	R	R	R	R			MDR
TB0207	R	EAI	R	R					MDR
TB035	R	EAI	R	R	R	R			MDR
TB062	R	EAI	R	R					MDR
TB1007	R	EAI	R	R					MDR
TB1023	R	LAM		R					–
TB1186	R	Beijing RD105	R	R					MDR
TB240	R	LAM	R						–
TB284	R	Beijing RD105	R	R	R		R		MDR
TB345	R	Beijing RD105	R	R					MDR
TB487	R	Beijing RD105	R	R					MDR
TB524	R	Beijing RD105		R					–
TB530	R	LAM	R	R	R				MDR
TB618	R	LAM				R		R	MDR
TB674	R	EAI	R	R					MDR
TB690	R	LAM	R	R					MDR
TB751	R	LAM				R			–
TB752	R	LAM	R	R	R				MDR
TB153	R	EAI	R	R					MDR

First-line TB drugs consist of isoniazid, rifampicin, pyrazinamide, ethambutol, Streptomycin [23]. Based on mutation analysis using TBProfiler on 19 samples of *M. tuberculosis* from Papua, resistance was found to first-line TB drugs such as rifampicin, isoniazid, streptomycin, ethambutol, and pyrazinamide. As Therapeutic Target Database (TTD) database provides annotations on the drug-protein interactions, it also annotates the second-line drug related to the SNP as well (Table 4). Resistance to rifampicin is caused by mutations in the *rpoB* gene [25]. In *M. tuberculosis* sample from Papua, several mutases were found in *rpoB* gene, namely S450L, D435Y, H445Y, L430P, Q432K (Table 4). These mutations have been confirmed to cause resistance to rifampicin [3, 26]^,^. In isoniazid, four genes were found containing mutations associated with isoniazid resistance, they were *katG* (S315T), *kasA* (G312S), *inhA* (I21V), and *fabG1*(C-15 T). Furthermore, TTD database also displayed that the protein of Bacterial Fatty acid synthetase I (Bact *inhA*) is the most common target for the first-line drug-related SNP as this is the most common finding in the wet laboratory. Resistance to streptomycin, ethambutol, and pyrazinamide was annotated respectively due to its mutations in the *rpsL* (K43R), *rrs* (A514C, A514T), *embB* (M306L, M306V, D1024N), and *pncA* (W119R) genes [25, 27].

**Table 4:** First-line and second-line drug-related SNPs identified using TBProfiler and TTD database search result of the drug functional annotations [24]

Drug	Gene	SNP mutation	Protein Target	TTD Link
First-line				
Rifampicin	*rpoB*	S450L, D435Y, H445Y, L430P, Q432K	Bacterial RNA polymerase switch region (Bact RNAP-SR)	http://db.idrblab.net/ttd/data/drug/details/d0g3dl
Isoniazid	*katG*	S315T	Bacterial Fatty acid synthetase I (Bact *inhA*)	http://db.idrblab.net/ttd/data/target/details/t79068
Isoniazid	*kasA*	G312S		
Isoniazid	*inhA*	I21V		
Isoniazid	*Rv1482c-fabG1*	C-15 T		
Streptomycin	*rpsL*	K43R	Staphylococcus 30S ribosomal subunit (Stap-coc pbp2)	http://db.idrblab.net/ttd/data/target/details/t72657
Streptomycin	*rrs*	A514C, A514T	Mycobacterium Arabinosyltransferase C (MycB *embC*)	http://db.idrblab.net/ttd/data/target/details/t30578
Ethambutol	*embB*	M306L, M306V, D1024N	Mycobacterium Arabinosyltransferase C (MycB *embC*)	http://db.idrblab.net/ttd/data/target/details/t30578
Pyrazinamide	*pncA*	W119R	Bacterial Fatty acid synthetase I (Bact *inhA*)	http://db.idrblab.net/ttd/data/target/details/t79068
Second-Line				
Amikacin	*rrs*	A514C	Staphylococcus 30S ribosomal subunit (Stap-coc pbp2)	http://db.idrblab.net/ttd/data/target/details/t72657

Second-line injectable drugs include kanamycin, amikacin, and capreomycin [28, 29]. Mutations in *rrs* gene are the cause of resistance to amikacin [25, 27]. The mutation generated from *M. tuberculosis* from Papua was A514C (Table 4). The application of WGS test in all cases of TB can assist TB control efforts and increase the accuracy of prediction in molecular resistance leading to more effective patient management and drug resistance surveillance [6, 9, 10]. This information is important for doctors to make quick decisions about the best therapy in the treatment of MDR-TB/XDR-TB [3, 5].

## Discussions

Since TB is one of diseases with highest burden in Papua Province of the Republic of Indonesia, thus, understanding the molecular epidemiology of *M. tuberculosis* Papua strain is very important. Sequencing technology, combined with bioinformatics and rapidly developing information technology, can be utilized in clinical fields such as public health microbiology [30]. Using NGS, WGS of *M. tuberculosis* can be completed faster, more affordable, and increasingly accessible as the alternative for molecular epidemiology studies. This database was supposed to contribute to the genetic polymorphism landscape of *M. tuberculosis* in Papua, especially the mutation that drives TB resistance. Moreover, genetic data will also enhance our understanding of strain distribution to see the cross-border transmission in borderline areas between two countries like Papua Province (Republic of Indonesia) and Papua New Guinea, especially in the area of Jayapura which is located in the borderline, and the interaction between people from Papua and Papua New Guinea are very intense. Therefore, this study assumed that the distribution of the strain is closely related to each other.

WGS on 19 samples of *M. tuberculosis* resulted in the locus coordinate of 4,354,210-4,396,040 bp; which was 99.6% similar to the first whole-genome sequence by Cole [5]. Cole revealed 4000 genes with very high guanine + cytosine content [5]. This size was slightly larger compared to *M. tuberculosis* PR08 from Malaysia. The size of the genome of *M. tuberculosis* PR08 was 4,292,364 bp with a G + C content of 65.2% [31]. Similarly, the genome of *M. tuberculosis* K strain from Korea had a size of 4,385,518 bp with a G + C content of 65.59%, 4194 CDSs, and 45 tRNAs, and one rRNA operon [32]. Meanwhile, TB751 from Papua had 25% in the subsystem consisting of 1080 CDS (987 non-hypothetical, 93 hypothetical) and 75% was not in the subsystem comprised with 3246 CDS (2177 non-hypothetical, 1069 hypothetical). Papua strain genomes were composed with 309 subsystems including 43 subsystems of virulence, disease, and defends. Besides carbohydrates and amino acids, Fatty Acids, lipids, and Isoprenoids dominated the subsystem (Fig. 1).

Furthermore, the most dominant CDS in the subsystem is amino acids and derivatives (26·85%), Carbohydrates (18.8%), Protein metabolism (16.86%), and cofactors, vitamins, prosthetics groups, and pigments (16.29%) (Fig. 1). In the non-tuberculous mycobacteria, the subsystem classification suggested that 18% of the gene ontologies belong to amino acids and its derivatives category, 12% to Cofactors, vitamins, prosthetic groups and pigments [33]. The biosynthesis of amino acids and their derivatives greatly determines the survival of bacteria. This function is controlled by the subsystems in the category of amino acids and their derivatives. Correspondingly, shikimate biosynthetic pathway is one of the controlled systems of this subsystem in which 3-deoxy-D-arabino- heptulosonate-7-phosphate (DAHP) will be converted into chorismite, which is useful in the synthesis of all aromatic amino acids, as well as other important metabolites in bacteria [34]. Vitamin B6, one of the most central molecules in the cells of living organisms, is an essential cofactor in various biochemical reactions that regulate primary cellular metabolism affecting overall physiology [35].

Meanwhile, RGPs can be the sites of integrated Mobile Genetic Elements (MGEs) insertions or the particular segments removal result of DNA in one or more TB strains. In TB751, two types of misc_RNA were found, but not in *M. tuberculosis* H37Rv, which were TB751_v1_miscRNA22 and TB751_v1_miscRNA23. Thus, the resulted product of the two regions is ASpks (Table 2). On the Mycobrowser server, Aspk is considered as a gene which produces putative small regulatory RNA whose function is still unknown.

Drug resistance profiling with WGS is very beneficial in detecting the specific mutations as well as in the correct interpretation to predict drug resistance or susceptibility [36].

Quality sequence data can be used to determine accurately the existing mutations associated with first-line and second-line drug resistance [2]. From the whole genome of *M. tuberculosis*, this investigation identified the mutations in genes associated with drug resistance in *M. tuberculosis* using TBProfiler [15]. TBProfiler gave 100% concordance with phenotypic DST results for INH, RIF, STR, ETB, ETH, and the fluoroquinolones [37]. In the case of resistance, this study analyzed 19 samples, 15 of which were MDR, and no sample was XDR [38]. However, the awareness of XDR should be improved because a Papuan strain resistant to amikacin (sample 618) had existed. The analysis revealed that MDR TB cases in Papua need to be monitored for the effectiveness of tuberculosis treatment. Overall, the analyzed samples displayed resistance to rifampicin, isoniazid, Ethambutol, streptomycin, pyrazinamide, and amikacinin (Table 3).

The mutations in *rpoB* gene has led the resistance to rifampin. Mutations at positions D435Y/V (D516Y/V), H445Y/D (H526Y/D), and S450L (S531L) (parentheses using *E. coli* residue numbering) have constituted the majority of mutations within this region [3, 39]. In the Papua strain, the mutations that were found were S450L, D435Y, H445Y, L430P, Q432K (Table 4). These mutations have been confirmed as the cause of resistance to rifampin through in vitro methods [27, 40]. Mutations in *katG*, *kasA*, *inhA*, and *fabG1* genes caused resistance to isoniazid. They encode Catalase-peroxidase-peroxynitritase T katG, beta-ketoacyl-ACP synthase, NADH- dependent enoyl-ACP reductase 3-Oxoacyl-[acyl-carrier-protein] reductase, and oxoacyl- [acyl-carrier protein] reductase fabG1. Genes mutation in that region has triggered *M. tuberculosis* resistant to isoniazid [41, 42]. Mutations in *katG*, *kasA*, *inhA*, and *fabG1* genes have provoked resistance to isoniazid. They encode Catalase-peroxidase-peroxynitritase T katG, beta-ketoacyl-ACP synthase, NADH- dependent enoyl-ACP reductase 3-Oxoacyl-[acyl-carrier-protein] reductase, and oxoacyl- [acyl-carrier protein] reductase fabG1. Genes mutation in that region have initiated *M. tuberculosis* resistant to isoniazid [43]. Meanwhile, any resistance to streptomycin can be induced by the mutases in *rpsL* (K43R) and *rrs* A514C, A514T genes [36]. In addition, any resistance to Ethambutol can occur when mutations appear in *embB* genes such as M306L, M306V, and D1024N occur [36]. Lastly, any resistance in pyrazinamide has occurred when there is a mutase in *pncA* gene, such as W119R [44].

Three lineages of *M. tuberculosis* strains of Papua were LAM, EAI, and Beijing. Beijing strain was reported as the responsible strain of MDR TB. In many areas of Asia, the Beijing strain was also reported in some cases to be the cause of TB outbreaks. Papua New Guinea reported Beijing strain and Euro-America as the lineage in Torres Strait that was riskily transported to Australia. In another part of PNG, particularly in Daru Island, an emergency of MDR-Tb by Beijing sub-lineage was reported. Using phylogenetic analysis combining with detailed molecular dating, they reveal that Beijing strains have been in local distribution since 1940 and first acquired drug resistance in 1960.

The comparison of the study finding in Papua Province of Indonesia with the previous finding in PNG can be defined as a cross border distribution of MTB. It is quite reasonable since the citizens of Papua Province travel between two countries daily for trading and other activities. Furthermore, the populations of the two regions were anthropologically connected. The other two lineages of Papua strain indicated that they were not too widespread among Asian countries. East African Indian was reported in Myanmar beside Beijing. LAM was found in Uganda but not found in another part of Indonesia. Therefore, in this open-access era, genomic surveillance of MTB is a necessary action for monitoring the strain mutation, especially on drug target genes and pathogenic associated genes [45].

The limitation of this study was the lack of epidemiology data describing TB patients’ characteristics because the samples were the unlinked specimens from the biobank of the provincial central laboratory. Moreover, the documentation of morphology and physiology of MTB was also minimal. However, this investigation had exposed the genetic data to define the potential surveillance approach of TD drugs resistance.

## Conclusion

The improvement of the genomic surveillance for the MTB strains in Papua are very important to comprehend the molecular epidemiology of this disease. The WGS data obtained can be used in monitoring the development of gene mutations associated with anti-TB drug resistance in Papuan strains. From the WGS data, it is known that most of the samples were MDR and no XDR cases were found.

## Methods

This investigation was a descriptive observational study using a molecular approach.

### Samples

Samples were selected randomly from *M. tuberculosis* archives of Papua Province referral laboratory. The total sample obtained was 20 *M. tuberculosis* from clinical sputum samples of non-HIV pulmonary TB patients. The diagnostic steps of TB clinical samples were carried out according to TB diagnostic standards using Indonesian national guidelines. The detection of *M. tuberculosis* was done by applying Ziehl Nielson.

Staining, MGIT test, and bacterial culture in Lowenstein Johnson medium. All specimens were checked by employing GeneX- pertMTB/RIF according to the Indonesian national guidelines.

### DNA extraction of *M. tuberculosis*

Deoxyribonucleic Acid (DNA) was extracted from bacterial colony on LJ medium by applying Qiaamp DNA Mini Kit (cat.51304). The procedure of DNA extraction was followed by the manufacturing instructions with some modifications. Before following the manual, the bacterial cell was boiled at 55^*o*^ C for 15 min. Then, the purity of the extraction results was measured by a nanodrop spectrophotometer at a wavelength of 260/280.

### Library preparation and sequencing

The quantification of extracted *M. tuberculosis* DNA was measured by employing Qubit TM 3.0 (Thermo Fisher Scientific). Next-Generation Sequencing procedure was then followed by Nextera XT DNA Library Prep Kit [46]. This NGS process was carried out using the MiSeq 600 cycle Reagent Kit (V3). WGS of *M. tuberculosis* was completed with NGS by applying MiSeq (Illumina Corp.) through the tagging of *M. tuberculosis* genome with Nextera transposon. Then, the library amplification was performed using Nextera PCR Master Mix, while the purification of the amplification product was conducted by applying AMPure XP beads. After purification, Nextera XT DNA Library Preparation Kits were utilized to normalize the library. Pooling DNA was finalized on a single tube and dilution was run using the Bead-Based Normalization Method [47]. Ultimately, DNA was loaded on the cartridge and WGS was performed on MiSeq.

### Bioinformatics pipeline

Genome analysis was performed by utilizing TBProfiler software https://github.com/jodyphelan/TBProfiler [14, 15] on Linux platform. Unipro Ugene NGS 1.3.1 (ugene net) was applied for BAM file analysis, visualization reading, consensus and alignment creation. *M. tuberculosis* genome was annotated using RAST Server (http://rast.theseed.org/FIG/rast.cgi) [12, 13]. Meanwhile, BLAST genome mapping was conducted using Gview server [18]. Genome analysis was completed by employing a Microscope server, especially on the Regions of Genomic Plasticity [11]. Furthermore, mycobacterium gene function was analyzed by Mycobrowser [48].

## Supplementary Information

**Additional file 1.**

## Data Availability

The datasets used and/or analyzed during the current study are available from the corresponding author on reasonable request.
